# Major Leslie R. Jordan

**Published:** 1944

**Authors:** 


### LESLIE ROLAND JORDAN, M.B., Ch.B. (Bristol), F.R.C.S., Major R.A.M.C.

Major Leslie Roland Jordan, R.A.M.C., met his death whilst operating in Normandy soon after the invasion landing. He qualified M.B., Ch.B. Bristol in 1931, and obtained his F.R.C.S. England in 1934. When war broke out he was practising in Muswell Hill and was Surgeon to the Hornsey Central Hospital. Mr. Wilfred Adams writes of him :—

Leslie R. Jordan was a born devotee of surgery. I enjoyed him as an enthusiastic student, valued his help as house surgeon at the Bristol Royal Infirmary and later his professional comradeship. Whilst still a resident at Southmead Hospital he made his first contribution to the literature with a report of a rare case of epitheliomatous invasion of the tibia from an overlying ulcer. He joined the Army in the early days of the war and was for a time stationed in the Orkney Isles. He went with the invading forces to Normandy and, when operating on casualties there, himself received a fatal wound. A sound clinician, he had a gently friendly way with patients and his life was one of unstinted service.

